# Atherogenic index of plasma is associated with major adverse cardiovascular events in patients with type 2 diabetes mellitus

**DOI:** 10.1186/s12933-021-01393-5

**Published:** 2021-10-05

**Authors:** Liyao Fu, Ying Zhou, Jiaxing Sun, Zhaowei Zhu, Zhenhua Xing, Shenghua Zhou, Yongjun Wang, Shi Tai

**Affiliations:** 1 Department of Blood Transfusion, The Second Xiangya Hospital of Central South University, Changsha, China; 2 Department of Cardiovascular Medicine, The Second Xiangya Hospital of Central South University, No. 139, Middle Renmin Road, Changsha, 410011 Hunan People’s Republic of China

**Keywords:** Atherogenic index of plasma, Type 2 diabetes mellitus, Cardiovascular disease, Major adverse cardiovascular events, Atherosclerosis, Prognosis

## Abstract

**Background:**

Previous studies reported the prognostic value of the atherogenic index of plasma (AIP) in the course of atherosclerosis and other cardiovascular diseases (CVDs). Still, the predictive utility of the AIP is unknown among patients with type 2 diabetes mellitus (T2DM).

**Methods:**

This was a secondary analysis of the Action to Control Cardiovascular Risk in Diabetes (ACCORD) study, which randomized 10,251 patients with long-lasting T2DM. ROC curve analysis was used to determine an optimal threshold for AIP, and the study population was divided into high and low AIP groups. Univariable and multivariable Cox proportional hazards regression analyses were used to determine the association between AIP and primary (major adverse cardiovascular events [MACEs], including nonfatal myocardial infarction, nonfatal stroke, and/or death from cardiovascular causes) and secondary outcomes (all-cause mortality). Stratified analyses were performed to control for the confounding factors.

**Results:**

AIP was an independent risk factor for the prognosis of T2DM (HR = 1.309; 95% CI 1.084–1.581; *P* = 0.005). The threshold for AIP was determined to be 0.34 in the study population. After adjustments for confounding factors, multivariable analysis showed that AIP was associated with the risk of MACEs (Model 1: HR = 1.333, 95% CI 1.205–1.474, *P* < 0.001; Model 2: HR = 1.171, 95% CI 1.030–1.333, *P* = 0.016; Model 3: HR = 1.194, 95% CI 1.049–1.360, *P* = 0.007), all-cause mortality (Model 1: HR = 1.184, 95% CI 1.077–1.303, *P* < 0.001), cardiovascular death (Model 1: HR = 1.422, 95% CI 1.201–1.683, *P* < 0.001; Model 3: HR = 1.264, 95% CI 1.015–1.573, *P* = 0.036), and nonfatal myocardial infarction (Model 1: HR = 1.447, 95% CI 1.255–1.669, *P* < 0.001; Model 2: HR = 1.252, 95% CI 1.045–1.499, *P* = 0.015; Model 3: HR = 1.284, 95% CI 1.071–1.539, *P* = 0.007). Subgroup stratified analyses showed that AIP might interact with sex, a classical risk factor of cardiovascular events.

**Conclusions:**

This study showed that AIP might be a strong biomarker that could be used to predict the risk of cardiovascular events in patients with T2DM.

*Trial registration:* URL: http://www.clinicaltrials.gov. Unique identifier: NCT00000620.

**Supplementary Information:**

The online version contains supplementary material available at 10.1186/s12933-021-01393-5.

## Introduction

Atherosclerotic cardiovascular disease (ASCVD) refers to a condition that involves cholesterol buildup in the arteries, often presenting as coronary heart disease, cerebrovascular disease, and peripheral artery disease of atherosclerotic origin. ASCVD is the leading cause of morbidity and mortality among individuals with diabetes globally, resulting in an estimated annual cost of $37.3 billion [1]. Type 2 diabetes mellitus (T2DM) has been associated with the early onset of ASCVDs [1]. Specifically, diabetic patients typically develop cardiovascular abnormalities with greater severity 14.6 years in advance than those without diabetes mellitus (DM) [2, 3]. Established risk factors for ASCVDs include hypertension and dyslipidemia, which are common in patients with T2DM [1]. Studies showed that patients pre-conditioned with dyslipidemia had dysregulated lipid and glucose metabolism (insulin resistance), resulting in a poorer prognosis of ASCVDs [4]. Although the incidence of T2DM complications has reduced over the years due to advances in medicine, more than 382 million people in the world currently have diabetes, making them more vulnerable to ASCVD-related disability and deaths [5]. Therefore, there is an urgent need for ASCVD prevention in diabetic individuals. In order to achieve this goal, it is necessary first to develop effective ways to predict and diagnose T2DM-related ASCVDs more accurately at early stages.

Various indices have been used to diagnose and prognosis of cardiovascular diseases (CVDs) alone [6]. For instance, the atherogenic index of plasma (AIP), a logarithmically transformed ratio of triglyceride (TG) to high-density lipoprotein-cholesterol (HDL-C) in molar concentration, was reported to be a sensitive marker of lipoprotein profiles [7]. Specifically, AIP could predict the size of lipoprotein particles, subsequently showing a positive correlation with the risk of CVDs [8, 9]. Furthermore, AIP can provide information on the severity of insulin resistance [10], which is associated with impaired glucose metabolism. Recently, AIP was reported as a novel independent prognostic biomarker of coronary artery disease [11–15] and arterial stiffness [16] beyond traditional risk factors.

Studies on T2DM showed that AIP was involved in major adverse cardiovascular events of T2DM, and a high AIP value might indicate a more severe form of T2DM [17–19]. A recent meta-analysis with 4010 patients suggested that AIP might be used as a simple, easy-to-calculate parameter in the prognosis of T2DM [20]. Moreover, diabetic patients with high AIP were reported to be at significantly higher risks for arterial stiffness and atherosclerosis [17]. Another study enrolled 2356 patients with T2DM and showed that AIP mainly affected the prognosis of T2DM after percutaneous coronary intervention, a procedure used to open blood vessels after the development of atherosclerosis [7]. Therefore, AIP might be a good indicator to predict the progression of T2DM in patients, especially after they were treated for blood vessel stenosis. Still, it is not known if AIP could be used as an even earlier prognostic marker for predicting the onset of atherosclerosis or other CVDs along with diabetes in individuals.

In this study, we speculated that patients with higher AIP might have a higher risk of developing major adverse cardiac events (MACEs) when accompanied by T2DM. The Action to Control Cardiovascular Risk in Diabetes (ACCORD) study was a clinical trial that originally aimed to study the effect of intensive glycemic control, intensive blood pressure control, and multiple lipid management in diabetic patients showing a high risk of CVD [21]. The ACCORD Follow-On Study (ACCORDION) was designed and conducted an additional follow-up of the participants [22]. We performed a secondary analysis on the data collected from ACCORD/ACCORDION and established three statistical regression models to rule out the confounding factors and assess the relationship between AIP and MACEs in T2DM patients with higher confidence. According to data analysis of the ACCORD study, the current study would establish a model of AIP, MACEs, and T2DM, which would be extremely useful in advancing the early detection and prognosis of cardiovascular events in diabetic patients.

## Methods

### Study population

We performed a secondary analysis on the published data of the ACCORD/ACCORDION trial (ClinicalTrials.gov number, NCT00000620) [21]. The rationale and design of the ACCORD trial have been described previously [21, 23, 24]. Briefly, ACCORD was a 2 × 2 factorial trial aiming to test whether strict control of blood glucose, blood pressure, and lipids could reduce the incidence of CVD in T2DM patients. The ACCORD closeout visits were completed by June 2009. Following approval by the coordinating center (Wake Forest University), participating clinical site institutional review board approval and consenting participants were invited to participate in the post-trial, nontreatment, observation-only ACCORDION study. Follow-up ended on October 31, 2014 (or 60 months post ACCORD), for a total of 5 years of post-trial observation [22]. In this randomized study, all 10,251 T2DM patients were recruited from 77 clinical sites across North America from January 2001 to October 2005. All individuals who participated in this study were T2DM patients between 40 and 79 years of age and who had glycosylated hemoglobin (HbA1c) levels of at least 7.5% and a history of CVD indicated by the anatomical evidence of significant atherosclerosis, albuminuria, left ventricular hypertrophy, or at least two risk factors for cardiovascular diseases. Tight control of blood pressure and lipids also did not reduce the risk of CVD. On the other hand, the intensive glycemic intervention was terminated after a mean follow-up of 3.7 years due to increased mortality in the intensive glycemia control group. All participants were transitioned to the standard glycemic control intervention. Follow-up continued for the remaining participants in the ACCORD trial.

### Data collection

The data included patients’ demographic and clinical characteristics, age, sex, ethnicity, education, smoking history, medical history, and previous medications, body measurements, blood content (i.e., plasma TG, cholesterol, LDL-C, and HDL-C), etc.

The primary outcome study was the occurrence of MACEs, including nonfatal myocardial infarction (MI), nonfatal stroke, and/or death from cardiovascular causes [25, 26]. The secondary outcome was all-cause mortality. The participants were followed up every 2–4 months by phone interviews or visits at the outpatient clinic. Relevant medical information was collected during each follow-up. The occurrence of MACEs in each patient was determined by a Working Group of the Morbidity and Mortality subcommittee. MACEs were collected when follow-up ended on October 31, 2014, or 60 months after ACCORD. All patients had a total of 5 years of post-trial observation.

### Definitions

The AIP is a logarithmically transformed ratio of TG to HDL-C in molar concentration (mmol/L), and it is mathematically derived from log (TG/HDL-C) [7]. Subsequently, all patients were divided into the high and low AIP groups according to the threshold determined by the receiver operating characteristic (ROC) curve analysis (presented in the next section).

### Statistical analysis

For the categorical variables, the baseline characteristics of the patients across the quartiles were defined in the form of frequencies and percentages. Chi-square tests were performed to analyze and compare the distributions of categorical variables. For continuous variables, the distribution was assessed by normal Q-Q plots. Depending on whether the datasets were normally distributed, either means and standard deviations (SDs) or median and interquartile ranges were used to describe the baseline characteristics. Normally distributed continuous variables were compared using one-way ANOVA. Mann–Whitney *U*-tests were performed otherwise.

A ROC curve was plotted to determine the optimal cut-off value of AIP for the prediction of MACE. The optimal cut-off value was determined according to the maximum Youden index, calculated as the sensitivity plus specificity minus one.

The relationship between AIP as a categorical variable and study outcomes was evaluated using Cox proportional hazard models. Then, multivariable models were used to adjust the associations according to confounding factors. Model 1: age (continuous variable), sex (categorical variable), previous cardiovascular event (categorical variable), smoking (categorical variable), BMI (continuous variable) and duration of diabetes (continuous variable). Model 2: age, sex, previous cardiovascular event, smoking, BMI, duration of diabetes, previous congestive heart failure (categorical variable), eGFR (continuous variable), HbA1c (continuous variable), plasma triglycerides (continuous variable), total plasma cholesterol (continuous variable), and plasma HDL-C (continuous variable). Model 3: age, sex, previous cardiovascular event, smoking, BMI, duration of diabetes, previous congestive heart failure, eGFR, HbA1c, plasma triglycerides, total plasma cholesterol, plasma HDL-C, insulin (categorical variable), biguanide (categorical variable), sulfonylurea (categorical variable), thiazolidinediones (categorical variable), statin (categorical variable), other lipid-lowering medications (categorical variable), niacin (categorical variable), and fibrate (categorical variable).

The Kaplan–Meier method provided a visual representation of survival over time, estimating the survival curves based on time-related events among patients. Subsequently, the survival curves were compared with log-rank tests. Stratified analyses were performed to test for interaction and control for confounding categorical variables including sex, age (< 65 or ≥ 65), race, history of cardiovascular disease, treatment, the trial involved, blood sugar concentration (HbA1C < 8.0% or ≥ 8.0%), as well as the incidence of depression. Cox regression was used for the subgroup analysis. If the interaction P-value was not significant, then the results of the different layers were consistent and reliable. If the interaction P-value was significant, it indicated a special population. Stata 15.1 (Stata Corp LLC, Texas, USA) was used to perform the statistical analyses. P < 0.05 was considered statistically significant for all analyses.

## Results

### Differences in baseline clinical characteristics among the MACE and non‑MACE groups of the study population

The demographics and clinical characteristics of the 10,251 T2DM patients are shown in Table 1. The patients were 62.81 (SD: 6.65) years of age. Among the 10,251 individuals, 61.45% were male, and 38.55% were female. Among all patients, 1826 patients (17.8%) developed MACEs after a median follow-up of 9.7 years.

**Table 1:** Characteristics of patients among the non-MACE and MACE group

Characteristics	Total (n = 10,251)	Non-MACE(n = 8425)	MACE (n = 1826)	*P*
Age, (years)	62.81 (SD: 6.65)	62.52 (SD: 6.51)	64.15 (SD: 7.13)	< 0.001
Sex (%)				< 0.001
Female	3952 (38.55)	3389 (40.23)	563 (30.83)	
Male	6299 (61.45)	5036 (59.77)	1263 (61.45)	
Living alone	8171 (79.72)	6735 (79.96)	1436 (78.64)	0.211
Race/ethnicity, n (%)				< 0.001
White	6393 (62.36)	5128 (60.87)	1265 (69.28)	
Non-white	3858 (37.64)	3297 (39.13)	561 (30.72)	
Education, n (%)				0.002
Less than high school	1521 (14.85)	1214 (14.42)	307 (16.84)	
High school graduate or GED	2704 (26.40)	2223 (26.40)	481 (26.39)	
Some college	3357 (32.77)	2740 (32.54)	617 (33.85)	
College degree or higher	2662 (25.99)	2244 (26.65)	418 (22.93)	
Previous cardiovascular event, n (%)	3609 (35.21)	2640 (31.34)	969 (53.07)	< 0.001
Previous congestive heart failure, n (%)	494 (4.82)	327 (3.88)	167 (9.15)	< 0.001
Previous hyperlipidemia, n (%)	7165 (69.90)	5862 (69.58)	1303 (71.36)	0.136
Previous hypertension, n (%)	7726 (75.37)	6301 (74.79)	1425 (78.04)	0.003
Cigarette-smoking status, n (%)				< 0.001
Current	1429 (13.94)	1146 (13.60)	283 (15.50)	
Former	4540 (44.29)	3664 (43.49)	876 (47.97)	
Never	4282 (41.77)	3615 (42.91)	667 (36.53)	
Weight (kg)	93.51 (SD: 18.41)	93.28 (SD: 18.40)	94.58 (SD: 18.40)	0.006
Body mass index (kg/cm^2^)	32.22 (SD: 5.40)	32.21 (SD: 5.41)	32.28 (SD: 5.37)	0.625
Blood pressure (mmHg)				
Systolic	136.36 (SD: 17.11)	136.00 (SD: 16.88)	138.02 (SD: 18.04)	< 0.001
Diastolic	74.88 (SD: 10.66)	75.14 (SD: 10.48)	73.70 (SD: 11.37)	< 0.001
Medications, n (%)				
Insulin	3260 (31.80)	2559 (30.37)	701 (38.39)	< 0.001
Metformin	6554 (63.94)	5467 (64.90)	1087 (59.53)	< 0.001
Any sulfonylurea	5474 (53.40)	4530 (53.77)	944 (51.70)	0.109
Any thiazolidinedione	2258 (22.03)	1912 (22.70)	346 (18.95)	< 0.001
ACEI/ARB	7102 (69.28)	5835 (69.26)	1267 (69.39)	0.933
Aspirin	5579 (54.68)	4538 (54.12)	1041 (57.26)	0.016
Statin	6500 (63.66)	5314 (63.33)	1186 (65.16)	0.147
Cholesterol absorption inhibitors	207 (2.03)	169 (2.02)	38 (2.09)	0.854
Niacin and nicotinic acid	183 (1.79)	142 (1.69)	41 (2.26)	0.118
Duration of diabetes (years)	10.80 (SD: 7.60)	10.50 (SD: 7.42)	12.18 (SD: 8.21)	< 0.001
Glycated hemoglobin (%)	8.30 (SD: 1.06)	8.28 (SD: 1.05)	8.41 (SD: 1.09)	< 0.001
Fasting plasma glucose (mg/dL)	175.19 (SD: 56.17)	174.04 (SD: 55.31)	180.51 (SD: 59.72)	< 0.001
Serum creatinine (mg/dL)	0.91 (SD: 0.23)	0.90 (SD: 0.23)	0.97 (SD: 0.25)	< 0.001
eGFR (mL/min/1.73 m^2^)				< 0.001
30–49 mL/min/1.73 m^2^	271 (2.64)	192 (2.28)	79 (4.33)	
> 50 mL/min/1.73 m^2^	9980 (97.36)	8233 (97.72)	1747 (95.67)	
Plasma triglycerides (mmol/L)	2.13 (SD: 1.68)	2.11 (SD: 1.65)	2.26 (SD: 1.81)	0.001
Total plasma cholesterol (mmol/L)	4.71 (SD: 1.13)	4.71 (SD: 1.12)	4.76 (SD: 1.19)	0.059
Plasma LDL-C (mmol/L)	2.70 (SD: 0.90)	2.69 (SD: 0.89)	2.74 (SD: 0.94)	0.024
Plasma HDL-C (mmol/L)	1.08 (SD: 0.31)	1.09 (SD: 0.31)	1.03 (SD: 0.31)	< 0.001
Atherogenic index of plasma (AIP)	0.54 (SD: 0.75)	0.51 (SD: 0.75)	0.64 (SD: 0.74)	< 0.001

Between patients who developed MACE and patients who did not, there was no significant difference in their living condition (living alone or not), history of hyperlipidemia, body mass index, and prescription record (i.e., sulfonylurea, ACEI/ARB, statin, cholesterol absorption inhibitors, or nicotinic acid). There were no marked differences in plasma cholesterol levels between the MACE and non-MACE groups, suggesting that it would not be a promising indicator to predict MACE. Compared with the non-MACE group, traditional risk factors for CVD, including old age, male, hypertension, and smoking, were more prevalent in diabetic patients with MACEs. Patients with MACEs also had significantly larger body weight, higher blood pressure, longer duration of diabetes, and higher incidence of cardiovascular events and congestive heart failure. In addition, they showed significantly higher levels of fasting plasma glucose, HbA1c, plasma TG, and LDL-C than non-MACE individuals. On the other hand, plasma HDL-C was lower in patients with MACEs than in those who did not develop MACEs. Subsequently, AIP, the marker for abnormal lipid and glucose metabolism and calculated as the ratio between TG and HCL-C on a logarithmic scale, was significantly higher in diabetic patients with MACEs than those without MACEs.

### The relationship between AIP and prognosis in patients with T2DM

In order to explore whether AIP was associated with the poor outcomes of diabetic patients, we obtained an optimal threshold of AIP that would best separate MACE and non-MACE individuals using ROC curve analysis. The results showed that AIP had an area under the curve (AUC) of 0.551 (95% CI 0.537–0.566), suggesting that there was an association between AIP and the risk of MACEs (Additional file 1: Figure S1). Furthermore, the optimal cut-off point for AIP was 0.34 according to the curve. The study population was assigned to two groups based on AIP: high AIP (greater than or equal to 0.34) and low AIP (less than 0.34).

**Fig. 1:**
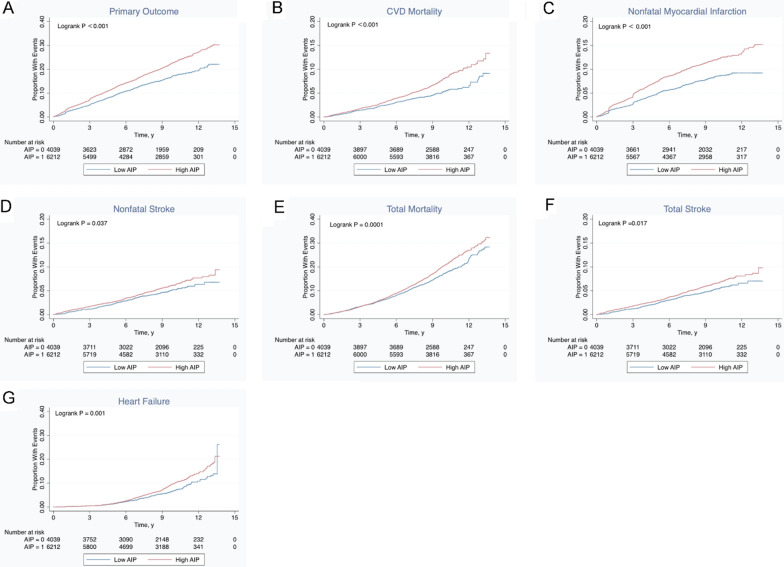
Kaplan–Meier curves for the primary and secondary outcomes. Low AIP vs. High AIP in **A** Primary outcome, **B** CVD mortality, **C** Nonfatal myocardial infarction, **D** Nonfatal stroke, **E** Total mortality, **F** Total Stroke, and **G** Heart failure

Next, AIP was assessed as a categorical variable using univariable Cox proportional hazards regression. During follow-up, 1233 patients with high AIP developed MACEs, while only 593 patients with low AIP had the same outcome (Table 2). Similar results were found in the analysis of the secondary outcomes: 1263 patients with high AIP died from any cause, while such poor outcomes were observed in 695 patients with low AIP (Table 2). AIP was an independent prognostic marker and associated with primary outcomes (HR: 1.383, 95% CI 1.254–1.525, *P* < 0.001) and secondary outcomes (all-cause death, HR: 1.205, 95% CI 1.099–1.322, *P* < 0.001) in T2DM patients with MACEs (Table 2). More specifically, high AIP presented the highest risk in cardiovascular deaths (HR: 1.500, 95% CI 1.270–1.765, *P* < 0.001) and nonfatal myocardial infarction (HR: 1.499, 95% CI 1.304–1.722, *P* < 0.001) (Table 2), suggesting that it could be used as a strong predictor of the two outcomes. Kaplan–Meier curves were used to visualize the probability of primary outcomes, the probability of specific cardiovascular events, including cardiovascular deaths, nonfatal myocardial infarction, nonfatal strokes (Fig. 1A–D), secondary outcomes, total strokes, and congestive heart failure (Fig. 1E–G). Compared with patients with low AIP, the probability of poor patient outcomes was significantly higher in the high AIP group (*P* < 0.05), further illustrating that AIP could be used as a good prognostic marker among patients with T2DM.

**Table 2:** Univariable Cox regression analysis of primary and secondary outcome

Outcomes	Total (n = 10,251)	Low AIP (n = 4039)	High AIP (n = 6212)	Univariable		
				HR	95% CI	P
Primary outcome	1826 (17.81)	593 (14.68)	1233 (19.85)	1.383	1.254–1.525	< 0.001
Cardiovascular cause death	669 (6.53)	205 (5.08)	464 (7.47)	1.500	1.270–1.765	< 0.001
Nonfatal myocardial infarction	936 (9.13)	287 (7.11)	649 (10.45)	1.499	1.304–1.722	< 0.001
Nonfatal stroke	488 (4.76)	171 (4.23)	317 (5.10)	1.219	1.012–1.468	0.037
Secondary outcomes (all-cause mortality)	1958 (19.10)	695 (17.21)	1263 (20.33)	1.205	1.099–1.322	< 0.001
Total stroke	516 (5.03)	178 (4.41)	338 (5.44)	1.248	1.041–1.496	0.017
Congestive heart failure	696 (6.79)	227 (5.62)	469 (7.55)	1.372	1.171–1.608	< 0.001

Data are expressed as HR and 95% CIs (reported in parentheses) as assessed by univariable cox regression analysis

Then, the hazard ratio of AIP for different patient outcomes was adjusted for confounding risk factors. Three multivariable regression models were established, each with a different number of confounders taken into consideration. Model 1 was adjusted for age, sex, history of cardiovascular events, smoking, BMI, and duration of diabetes; AIP showed a hazard ratio of 1.333 for MACEs (95% CI 1.205–1.474, *P* < 0.001) and a lower hazard ratio of 1.184 for all-cause mortality (95% CI 1.077–1.303, *P* < 0.001) (Table 3). Model 2 was adjusted for additional variables on top of Model 1, including a history of congestive heart failure, eGFR, HbA1c, plasma TG, total plasma cholesterol, and plasma HDL-C. Model 3 was based on Model 2, with additional confounders in regard to prescription records, including the use of insulin, biguanide, sulfonylurea, thiazolidinediones, statin, other lipid-lowering medications, niacin, and fibrate. The association between AIP and MACEs remained significant, with a hazard ratio of 1.171 under model 2 and 1.194 under model 3 (Model 2: 95% CI 1.030–1.333, *P* = 0.016; Model 3: 95% CI 1.049–1.360, *P* = 0.007) (Table 3). Association was also observed between AIP and cardiovascular deaths or nonfatal myocardial infarction (Table 3). However, after adjustment for confounders, AIP showed hazard ratios less than one for nonfatal stroke, total stroke, congestive heart failure, and all-cause mortality, representing weak or no association (Table 3).

**Table 3:** Multivariable Cox regression analysis of primary and secondary outcomes

Outcome	Model 1			Model 2			Model 3		
	HR	95% CI	P	HR	95% CI	P	HR	95% CI	P
Primary outcome (MACEs)	1.333	1.205–1.474	< 0.001	1.171	1.030–1.333	0.016	1.194	1.049–1.360	0.007
Cardiovascular cause death	1.422	1.201–1.683	< 0.001	1.237	0.995–1.538	0.056	1.264	1.015–1.573	0.036
Nonfatal myocardial infarction	1.447	1.255–1.669	< 0.001	1.252	1.045–1.499	0.015	1.284	1.071–1.539	0.007
Nonfatal stroke	1.190	0.984–1.441	0.073	1.078	0.841–1.381	0.590	1.090	0.849–1.399	0.680
Secondary outcomes (all-cause mortality)	1.184	1.077–1.303	< 0.001	1.037	0.917–1.173	0.559	1.065	0.942–1.206	0.315
Total stroke	1.232	1.023–1.484	0.028	1.132	0.888–1.444	0.316	1.143	0.895–1.459	0.284
Congestive heart failure	1.264	1.074–1.487	0.005	1.035	0.840–1.276	0.746	1.017	0.823–1.255	0.879

Data are expressed as HR and 95% CIs (reported in parentheses) as assessed by multivariable Cox regression analysis; HR: hazard ratio; CI: confidence interval. Covariables included in multivariable cox regression models were model 1: age, sex, previous cardiovascular event, smoking, BMI, and duration of diabetes. Model 2: age, sex, previous cardiovascular event, smoking, BMI, duration of diabetes, previous congestive heart failure, eGFR, HbA1c, plasma triglycerides, total plasma cholesterol, and plasma HDL-C. Model 3: age, sex, previous cardiovascular event, smoking, BMI, duration of diabetes, previous congestive heart failure, eGFR, HbA1c, plasma triglycerides, total plasma cholesterol, plasma HDL-C, insulin, biguanide, sulfonylurea, thiazolidinediones, statin, other lipid-lowering medications, niacin, and fibrate

### The association between AIP and MACEs in the different subgroups of the study population

Next, in order to explore the association between AIP and MACEs in more detail, we categorized the study population based on patient demographics and medical records, including sex, age, race/ethnicity, history of cardiovascular diseases (CVD), treatment given, trial, HbA1c levels, and incidence of depression. Subsequently, stratified analyses were performed to test for interactions and stratified confounders in the association between AIP and MACEs in the different subgroups (Table 4, Additional file 1: Figure S2). The results showed that sex might play a role in the association between AIP and MACEs, leading to a stronger prediction of MACEs by AIP among women. However, we did not detect any interaction among different demographic factors and clinical records in male patients. On the other hand, sex seemed to also interact with the association between AIP and nonfatal myocardial infarction. Taken together, the stratified analyses suggested that AIP might be a stronger prognostic marker among elderly women. Furthermore, the association between AIP and nonfatal myocardial infarction was evaluated, and similar results were found across different population subgroups (Table 4, Additional file 1: Figure S3).

**Table 4:** Hazard ratios for the primary outcome and death from any Cause in prespecified Subgroups

Outcome	Low AIP		High AIP		HR^a^	95% CI	P	P for interaction^b^
	Events/n	%	Events/n	%				
MACEs								
Sex								0.024
Male	389/2207	17.63	874/4092	21.36	1.230	1.091–1.386	0.001	
Female	204/1832	11.14	359/2120	16.93	1.566	1.318–1.859	< 0.001	
Age								0.912
< 65	298/2416	12.33	690/4073	16.94	1.426	1.245–1.634	< 0.001	
≥ 65	295/1623	18.18	543/2139	25.39	1.410	1.224–1.625	< 0.001	
Race/ethnicity								0.557
White	336/1987	16.91	929/4406	21.08	1.286	1.135–1.457	< 0.001	
Non-white	257/2052	12.52	304/1806	16.83	1.368	1.159–1.615	< 0.001	
CVD history								0.254
Yes	300/1260	23.81	669/2349	28.48	1.241	1.083–1.422	0.002	
No	293/2779	10.54	564/3863	14.60	1.391	1.208–1.602	< 0.001	
Glycemia arm								0.716
Standard	301/2021	14.89	629/3102	20.28	1.408	1.227–1.615	< 0.001	
Intensive	292/2018	14.47	604/3110	19.42	1.358	1.181–1.562	< 0.001	
Trail								0.131
BP	311/2295	13.55	468/2438	19.20	1.473	1.277–1.700	< 0.001	
Lipid	282/1744	16.17	765/3774	20.27	1.263	1.102–1.448	0.001	
HbA1c								0.072
< 8.0	243/1971	12.33	548/2898	18.91	1.553	1.335–1.806	< 0.001	
≥ 8.0	350/2068	16.92	685/3314	2067	1.260	1.108–1.433	< 0.001	
Depression								0.257
Yes	122/802	15.21	363/1619	22.42	1.519	1.238–1.865	< 0.001	
No	470/3235	14.53	870/4593	18.94	1.325	1.184–1.482	< 0.001	
Nonfatal myocardial infarction								
Sex								0.064
Male	190/2207	8.61	457/4092	11.17	1.318	1.112–1.561	0.001	
Female	97/1832	5.29	192/2120	9.06	1.747	1.369–2.230	< 0.001	
Age								0.761
< 65	146/2416	6.04	373/4073	9.16	1.566	1.293–1.896	< 0.001	
≥ 65	141/1623	8.69	276/2139	12.90	1.498	1.223–1.835	< 0.001	
Race/ethnicity								0.648
White	170/1987	8.56	501/4406	11.37	1.362	1.144–1.621	< 0.001	
Non-white	117/2052	5.7	148/1806	8.19	1.462	1.147–1.864	0.002	
CVD history								0.158
Yes	152/1260	12.06	356/2349	15.16	1.287	1.064–1.555	0.009	
No	135/2779	4.86	293/3863	7.58	1.572	1.282–1.928	< 0.001	
Glycemia arm								0.891
Standard	151/2021	7.47	341/3102	10.99	1.513	1.249–1.832	< 0.001	
Intensive	136/2018	6.74	308/3110	9.90	1.485	1.214–1.817	< 0.001	
Trail								0.025
BP	143/2295	6.23	257/2438	10.54	1.747	1.424–2.144	< 0.001	
Lipid	144/1744	8.26	392/3774	10.39	1.266	1.046–1.532	0.016	
HbA1c								0.600
< 8.0	112/1779	6.30	255/2602	9.80	1.568	1.255–1.958	< 0.001	
≥ 8.0	175/2260	7.74	394/3610	10.91	1.450	1.214–1.733	< 0.001	
Depression								0.203
Yes	61/802	7.61	208/1619	12.85	1.729	1.300–2.300	< 0.001	
No	226/3235	6.99	441/4593	9.60	1.394	1.188–1.637	< 0.001	

^a^Low AIP as a reference, the Hazard Ratio of High AIP for the primary outcome or nonfatal myocardial infarction in each subgroup in sex, age, race/ethnicity, CVD history, glycemia arm, trial, HbA1c, and depression

^b^Interaction between categorical factor AIP and sex, age, race/ethnicity, CVD history, glycemia arm, trial, HbA1c, and depression, respectively

## Discussion

In this retrospective analysis of T2DM patients with high CVD risk, AIP was a parameter related to abnormal lipid and glucose metabolism. The occurrence of MACEs of the high AIP group was significantly higher than that of the low AIP group. These differences were mainly caused by cardiovascular death and non-fatal myocardial infarction. In the subgroup analysis, we found that AIP has a consistent effect on the prognosis of T2DM patients. Therefore, AIP can be used as a predictor of the long-term prognosis of patients with T2DM.

Currently, diabetes is affecting hundreds of million people’s health and living conditions, with a global prevalence of roughly 9.3% [27]. In 2019, it was reported that diabetes directly accounted for about 1.5 million deaths [27]. ASCVDs, or diseases that involve cholesterol buildup in the arteries, are the leading cause of morbidity and mortality among individuals with diabetes in the world [1]. Diabetic patients would typically develop more severe ASCVDs and at an earlier age compared with non-diabetic individuals [2, 3]. Therefore, it is critical to identify an effective prognostic and diagnostic marker to enhance preventive care in high-risk individuals. Originally, the Action to Control Cardiovascular Risk in Diabetes (ACCORD) trial studied 10,251 randomized patients with long-standing T2DM, aiming to understand the effect of intensive glycemic control, intensive blood pressure control, and multiple lipid management in preventing ASCVD [21]. Previously, AIP, a parameter that measures lipid and glucose metabolism, has been considered a prognostic marker for CVD [6]. A recent study also revealed that AIP could be associated with body fat levels in T2DM patients [28]. However, few studies investigated the relationship between AIP and MACEs in T2DM patients. In our post hoc analysis of the ACCORD trial, we found that the AIP plays an important role in the long-term prognosis of T2DM patients. By analyzing the AIP of the 10,251 T2DM patients, the optimal cut-off value for AIP was 0.34. Patients with AIP higher than 0.34 were at a significantly higher risk of developing MACEs than those in the low AIP group. More specifically, AIP showed greater prognostic power in predicting cardiovascular deaths and nonfatal myocardial infarction. Furthermore, the study population was subcategorized based on demographic and clinical parameters, and AIP had consistent effects in predicting patient outcomes in different subgroups.

Compared with patients without T2DM, patients with T2DM tend to have more cardiovascular risk factors, including hyperlipidemia. Still, previous studies reported no significant differences in LDL-C levels between diabetic and nondiabetic patients [29]. Moreover, LDL-C was not a promising indicator of poor prognosis among diabetic patients [8]. On the other hand, AIP, or the ratio between TG to HDL-C on a logarithmic scale, quantified one’s ability to metabolize glucose and lipid and was found to be independent of LDL-C [7]. More specifically, AIP was shown to be a more promising predictor of atherosclerosis than LDL-C levels [8, 30] and could be used in addition to the traditional risk factors [9, 12].

The present study confirmed that AIP could be used as an independent predictor for the prognosis of T2DM patients in the long-term follow-up, suggesting that it could more accurately reflect the comprehensive situation of lipids and glucose metabolism among diabetic patients. Consistent with our findings, Zheng et al. [7] also reported that high AIP indicated a higher risk of MACEs among diabetic patients after percutaneous coronary intervention (PCI) in a single-center observational cohort study. Moreover, AIP might also be a powerful complementary index to assess cardiometabolic risks in children and adolescents [31]. In addition, other studies suggested that AIP was also a simple and useful tool in identifying insulin-resistant patients at higher cardiometabolic risk [32], more effective than the visceral adiposity index, which was used traditionally [33]. Overall, AIP is an independent clinical marker critical to the prognosis of cardiovascular events in a different subpopulation, including patients with T2DM.

Previous studies showed that the mean values of AIP ranged from -0.24 to 0.55 in the general population [8]. AIP can be divided into three different ranges, each representing a level of risk for CVD: AIP < 0.11 (low risk), 0.11 < AIP < 0.21 (intermediate risk), and AIP > 0.21 (high risk) [34, 35]. Meanwhile, patients with T2DM have higher AIP than the general population; as a result, this study used different cut-off values for AIP in our analysis. The optimal cut-off point for AIP was 0.34 among patients with T2DM, which aligned better with another study (n = 2356) that used 0.318 as the threshold for analysis; the slight difference could be due to the size difference in the study population [7].

AIP correlates to lipoprotein particle size and could be used as a marker for plasma atherogenicity [8]. Here we reported that the prognosis of the high AIP group was significantly worse than that of the low AIP group, and the difference was mainly due to MACEs, including cardiovascular deaths and nonfatal myocardial infarction. From these results, we speculated that AIP was most likely a reflection of atherosclerosis, the primary underlying cause of CVD that can lead to stroke and acute coronary syndrome. AIP was thereby associated with acute coronary syndromes, CVD, and its risk factors [8, 30]. In patients with T2DM, the incidence of MACEs was lower in patients with lower AIP after PCI, possibly because of the low rate of revascularization [7]. Therefore, AIP could be a powerful marker for cardiovascular events in patients with T2DM, and further research is needed to unveil the molecular mechanism behind the correlation between AIP, MACEs, and T2DM.

This study has some limitations. First, this was a post hoc, exploratory analysis of the ACCORD trial, and there might be confounding factors included in the original study that we could not control for. Second, the patients included in the study were mainly Caucasians; subsequently, our conclusions might not apply to other populations. Despite that these limitations might interfere with the clinical application of the AIP threshold found in our study, our results have shown that it is absolutely necessary to strictly manage lipid and glucose levels in T2DM patients. Third, parameters used to calculate AIP was collected during the study, and changes in AIP were not monitored during follow-up. Hence, further studies are needed to evaluate the clinical application of AIP among patients with T2DM.

## Conclusion

By analyzing a large-scale clinical trial that involved 10,251 randomized T2DM patients, this study suggested that AIP could be a strong prognostic marker to assess the risk of cardiovascular events in patients with T2DM. Specifically, diabetic patients with high AIP were more likely to experience MACEs. The information obtained from this study has provided more insights on the discovery and clinical guidance of a new MACE bioindicator to be used among high-risk populations.

## Supplementary Information

**Additional file 1: Figure S1.** Area Under the Curve (AUC) for AIP in differentiating MACEs outcome in the cohort. **Figure S2**. Risk of major adverse cardiovascular events. **Figure S3**. Risk of nonfatal myocardial infarction.

## Data Availability

The datasets used and analyzed during the current study are available from the ACCORD/ACCORDION Research Materials obtained from the National Heart, Lung, and Blood Institute (NHLBI) Biologic Specimen and Data Repository Information Coordinating Center. The contents of this report do not necessarily reflect the opinions or views of the ACCORD/ACCORDION study authors or the NHLBI.
