# Supplementary material for: Atherogenic index of plasma is associated with major adverse cardiovascular events in patients with type 2 diabetes mellitus
Source: Cardiovasc Diabetol. 2021 Oct 5;20:201. doi: 10.1186/s12933-021-01393-5 (PMC8493717; doi:10.1186/s12933-021-01393-5)
Supplement: Supplementary file 1 — Additional file 1: Figure S1. Area Under the Curve (AUC) for AIP in differentiating MACEs outcome in the cohort. Figure S2. Risk of major adverse cardiovascular events. Figure S3. Risk of nonfatal myocardial infarction. [file 12933_2021_1393_MOESM1_ESM.docx]

**Additional file 1:**


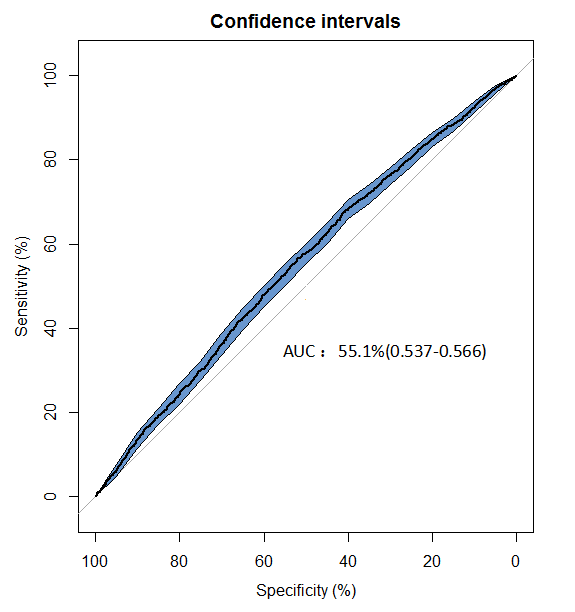


**Figure S1.** Area Under the Curve (AUC) for AIP in differentiating MACEs outcome in the cohort.


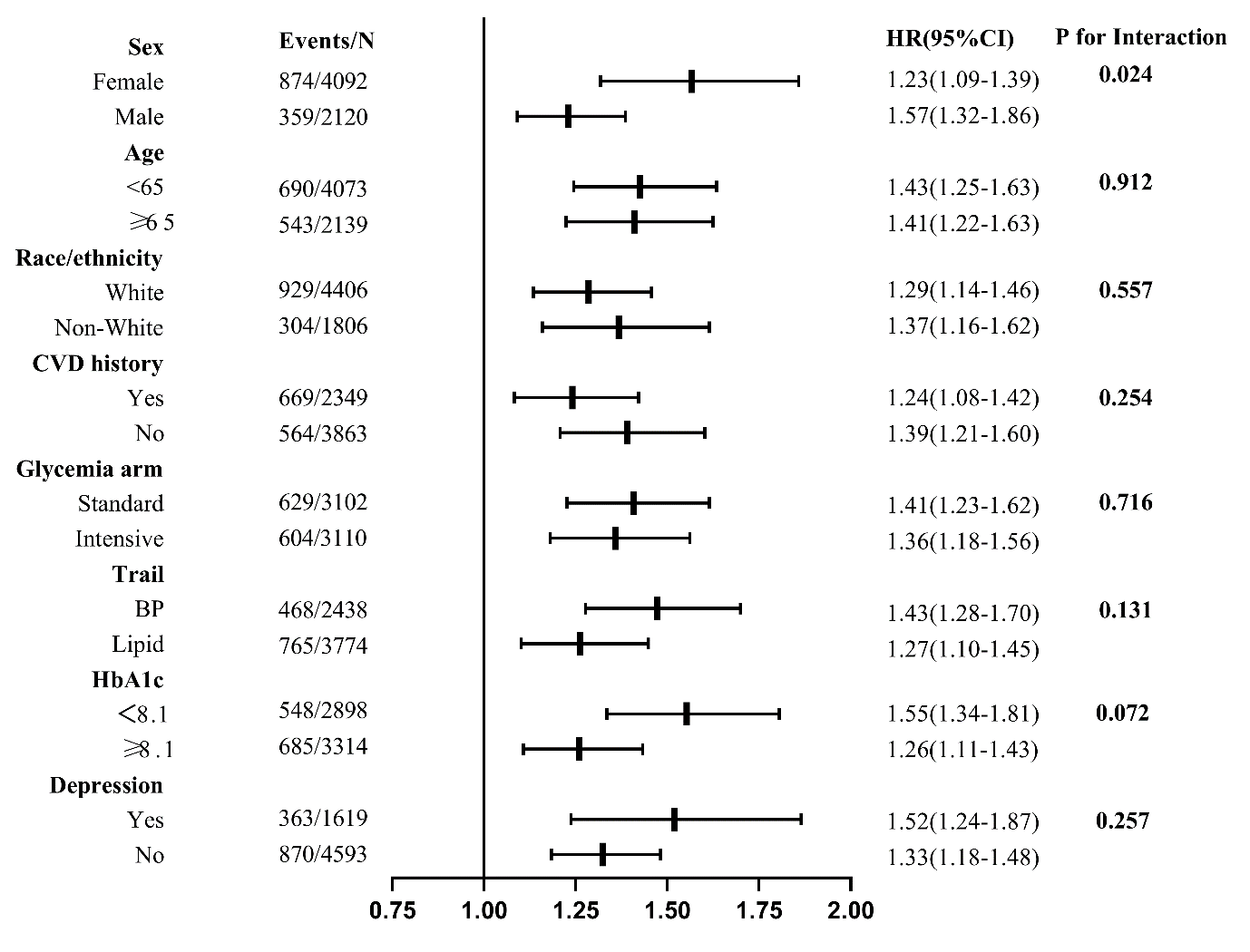


**Figure S2**. Risk of major adverse events.


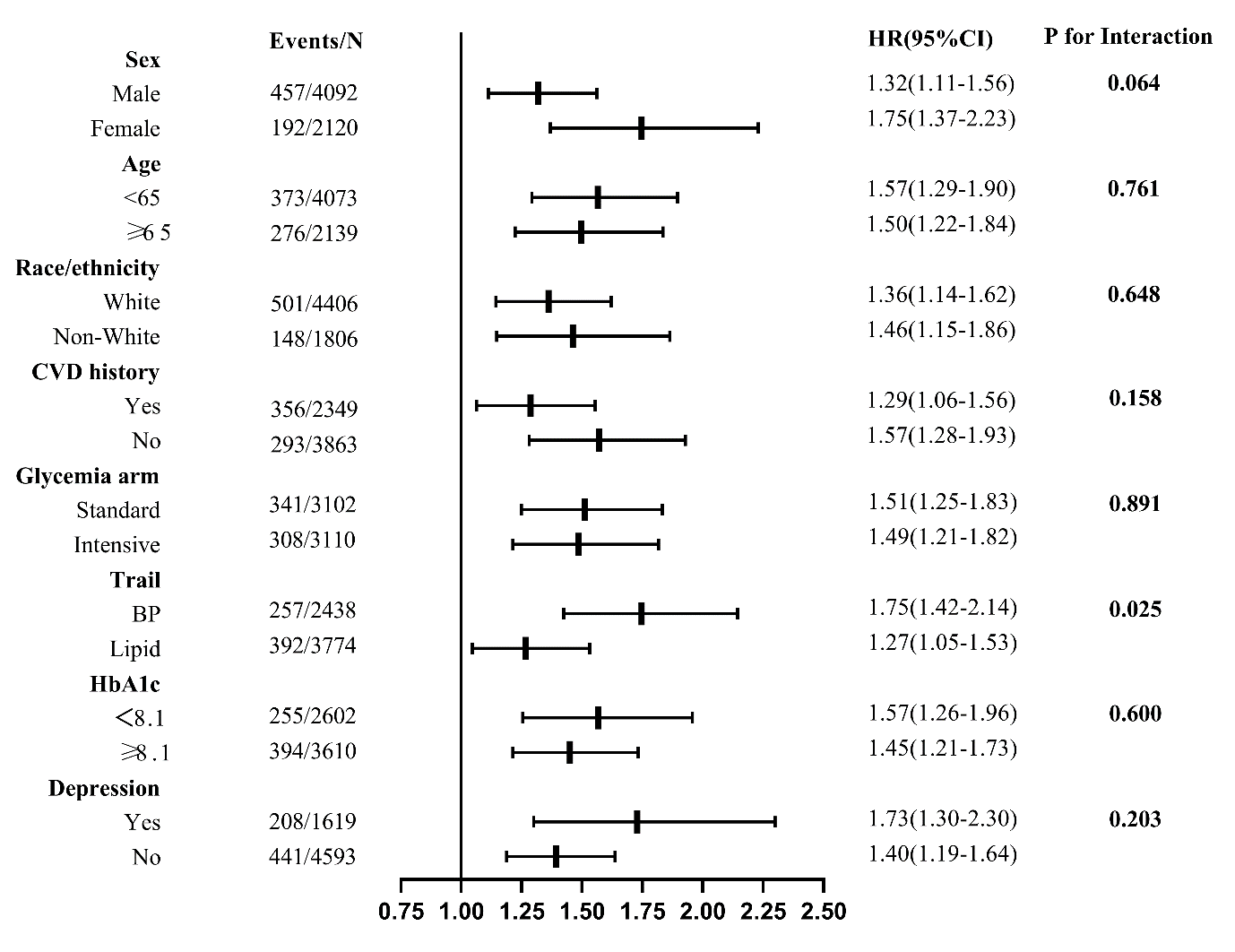


**Figure S3**. Risk of Nonfatal myocardial infarction.
